# Positive association between LDH to albumin ratio and arthritis: A cross-sectional NHANES study (1999–2010)

**DOI:** 10.1097/MD.0000000000048096

**Published:** 2026-03-27

**Authors:** Meilin Wu, Conghai Liu, Meng Zheng, Zhicheng Wei, Xiaodong Sun

**Affiliations:** a Department of Clinical Pharmacy, Dazhou Central Hospital, Dazhou, Sichuan Province, People’s Republic of China; b Department of Radiology Diagnosis, The Fifth Medical Center of Chinese PLA General Hospital, Beijing, People’s Republic of China.

**Keywords:** arthritis, inflammation, lactate dehydrogenase to albumin ratio, metabolic status, nutritional status

## Abstract

Arthritis is a prevalent condition posing a significant threat to human health. The lactate dehydrogenase to albumin ratio (LAR) has been recognized as a key indicator for the onset and progression of various diseases. However, its association with arthritis remains incompletely investigated. This cross-sectional study analyzed data from adult participants in the National Health and Nutrition Examination Survey (1999–2010). Arthritis identification was based on self-reported information. We employed weighted logistic regression analysis to assess the odds ratio (OR) and 95% confidence interval (CI) for the association between log_2_-transformed LAR (log_2_(LAR)) and arthritis. We also employed subgroup and sensitivity analyses to evaluate the robustness of results. Among 8616 participants, 2229 had arthritis. After full covariate adjustment, log_2_(LAR) was significantly associated with arthritis (OR: 1.52; 95% CI: 1.16–2.01; *P* = .003). Participants in the highest log_2_(LAR) quartile had a 37% higher arthritis risk versus the lowest quartile (adjusted OR: 1.37; 95% CI: 1.07–1.74; *P* = .013). Subgroup and sensitivity analyses confirmed result stability. Elevated log_2_(LAR) levels were associated with increased risk of arthritis (OR: 1.52; 95% CI: 1.16–2.01; *P* = .003).

## 1. Introduction

Arthritis, a prevalent musculoskeletal disorder, is characterized by joint inflammation, pain, and reduced mobility.^[1,2]^ Its pathogenesis involves diverse mechanisms, including autoimmune responses, joint degeneration, and metabolic imbalances.^[3,4]^ This disorder encompasses a wide spectrum of subtypes, such as rheumatoid arthritis, osteoarthritis, and gout.^[2,5]^ Arthritis affects a significant proportion of the global population, with prevalence increasing substantially with age.^[1,6]^ It has emerged as a leading cause of disability, imposing a heavy burden on individuals, families, and society at large.^[7]^ Therefore, early detection and effective management of arthritis are crucial for improving patients’ quality of life and reducing the economic impact.

Inflammation and nutritional status play key roles in the development and progression of arthritis.^[8]^ Lactate, a metabolite produced during anaerobic glycolysis, can reflect the body’s metabolic and inflammatory state.^[9,10]^ Albumin, a negative acute-phase protein with anti-inflammatory properties, serves as a key indicator of nutritional status.^[11,12]^ The lactate dehydrogenase to albumin ratio (LAR), integrating both markers, represents a potential biomarker reflecting both inflammatory activity and nutritional status.^[13]^ The lactate dehydrogenase to albumin ratio, identified as a potential biomarker, has been extensively studied as a prognostic indicator in various conditions, including cancer, liver conditions, pulmonary embolism, infection-related kidney injury, and so on.^[13-17]^ This marker shows potential for prognostic assessment across a range of medical conditions. However, there is a significant gap in research regarding the association between LAR and arthritis. Exploring this association could improve understanding of arthritis mechanisms and facilitate the early identification of high-risk individuals. Therefore, this cross-sectional study utilized NHANES data (1999–2010) to examine the association between LAR and arthritis risk. We hypothesized that elevated LAR levels are associated with increased arthritis susceptibility.

## 2. Materials and methods

### 2.1. Study population

The NHANES database, conducted by the National Center for Health Statistics (NCHS), evaluates the health and nutritional status of the US population.^[18]^ It employs a stratified, multistage probability sampling technique to ensure national representativeness.^[19]^ The survey involves an in-home interview followed by a standardized medical examination at a Mobile Examination Center. The NCHS Research Ethics Review Board granted ethical approval, and all participants provided written informed consent. Further details are accessible via the NCHS website. Secondary analysis of publicly available, de-identified NHANES data requires no additional Institutional Review Board (IRB) approval.^[20]^

**Figure 1.**
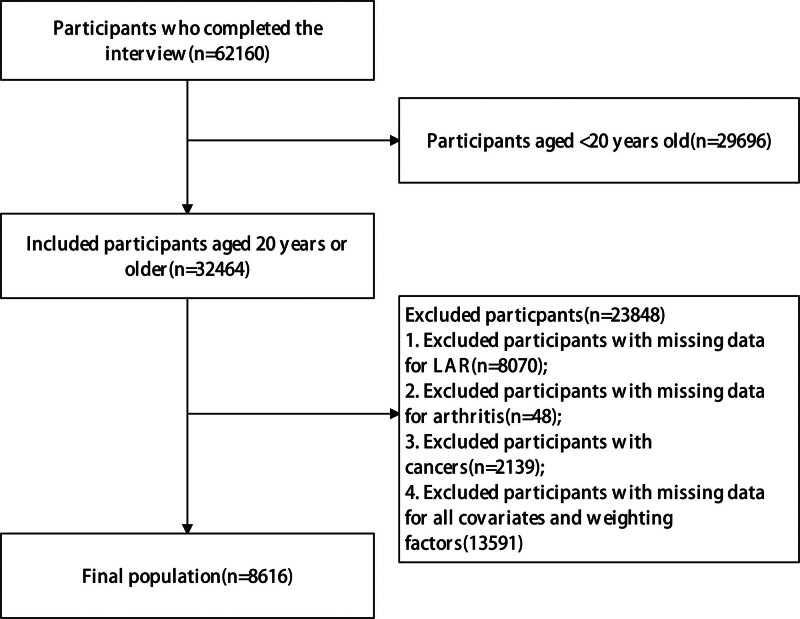
Flowchart of participants selection. LAR = lactate dehydrogenase to albumin ratio.

This retrospective cross-sectional study utilized NHANES data from 1999 to 2010. We excluded participants aged < 20 years, or had incomplete data on lactate dehydrogenase, albumin, arthritis status, key covariables, or sampling weights. Figure 1 illustrates the participant selection flowchart, detailing the application of exclusion criteria.

### 2.2. Assessment of lactate dehydrogenase to albumin ratio

In this study, LAR was calculated as lactate dehydrogenase (LDH) level (International Units per liter, IU/L) divided by albumin concentration (grams per liter, g/L). Specimens were collected at the Mobile Examination Center and underwent processing in a certified laboratory. NHANES implemented rigorous quality control protocols throughout data collection and laboratory analysis to ensure result accuracy and reliability. Comprehensive documentation regarding laboratory methodology, quality assurance procedures, and monitoring is available through the NHANES website: https://wwwn.cdc.gov/nchs/nhanes/analyticguidelines.aspx.

### 2.3. Assessment of arthritis

Medical status was assessed through a standardized questionnaire administered by trained healthcare professionals, including physicians. Each participant was asked: “Has a doctor or other health professional ever told you that you had arthritis?” Individuals responding “yes” were classified as having arthritis, whereas those responding “no” were considered arthritis-free. Participants who answered affirmatively were further queried: “Which type of arthritis was it?” Based on their responses, they were subcategorized as having rheumatoid arthritis, osteoarthritis, or other specified types.^[21]^

### 2.4. Covariates

In this study, covariate selection was based on clinical relevance and findings from prior studies.^[21-24]^ Potential covariates included demographics: sex, age, race/ethnicity (non-Hispanic White, non-Hispanic Black, Mexican American, Other), marital status (living independently [never married, separated, divorced, widowed], living with partner [married, cohabiting]), education (<9 years, 9–12 years, >12 years).^[25]^ Socioeconomic status: family income-to-poverty ratio (PIR: low [≤1.3], medium [>1.3 to ≤3.5], high [>3.5]).^[26]^ Lifestyle factors: smoking status (never, former, current),^[25,26]^ alcohol consumption (nondrinker [<12 lifetime drinks], former drinker [≥12 drinks/yr historically, none in past year], current drinker [≥12 drinks/yr and drank in past year]),^[26]^ physical activity level (sedentary, moderate, vigorous). Anthropometric and clinical measures: body mass index (BMI: <25 kg/m^2^, ≥30 kg/m^2^), hypertension (self-reported physician diagnosis), hyperlipidemia (total cholesterol ≥ 5.18 mmol/L, triglycerides ≥ 150 mg/dL, high-density lipoprotein cholesterol < 1.30 mmol/L [women]/<1.04 mmol/L [men], low-density lipoprotein cholesterol ≥ 3.37 mmol/L),^[27,28]^ diabetes (self-reported physician diagnosis), cardiovascular disease (CVD; self-reported congestive heart failure, myocardial infarction, coronary artery disease, angina pectoris, or stroke),^[21,27]^ chronic kidney disease (CKD; eGFR < 60 mL/min/1.73 m^2^ or urinary albumin-to-creatinine ratio > 30 mg/g; eGFR calculated via CKD-Epidemiology Collaboration creatinine equation).^[28]^ Laboratory measures: C-reactive protein (CRP), white blood cell count (WBC), alanine aminotransferase (ALT), aspartate aminotransferase (AST; all measured via laboratory testing). Dietary intake: vitamin C intake, zinc intake (obtained from dietary interviews). Self-reported physician diagnosis defined preexisting hypertension, diabetes, and CVD. Comprehensive variable documentation is available on the NHANES website: https://wwwn.cdc.gov/nchs/nhanes/Default.aspx.

### 2.5. Statistical analysis

When analyzing the NHANES dataset, It is importance to integrate sampling weights and design variables. This practice helps prevent biased estimates and inflated significance levels.^[29]^ Following NHANES analytical guidelines, we accounted for the complex survey design using masked variance pseudo-stratum (SDMVSTRA), masked variance pseudo-primary sampling unit (SDMVPSU), fasting subsample weights, WTSAF2YR (2-year) and WTSAF4YR (4-year).^[29]^ The WTSAF4YR was specifically employed for data encompassing NHANES cycles 1999 to 2000 and 2001 to 2002. The sampling weights for the 1999 to 2010 period were calculated as follows: for the years 1999 to 2002, the weights were set at one-third of the WTSAF4YR value. For the subsequent cycles, the weights were determined as one-sixth of the WTSAF2YR value. This approach ensures that the study’s findings are representative and statistically robust.^[26,30]^

The sociodemographic, clinical, laboratory, and dietary characteristics of all participants were summarized across quartiles of log_2_(LAR). Categorical variables were shown as unweighted counts and their weighted percentages. On the other hand, continuous variables were shown as averages and their standard errors. To compare differences among groups within the complex NHANES survey design, we applied Rao & Scott’s second-order corrected chi-square tests for categorical variables and Wilcoxon rank-sum tests for continuous variables. All analyses incorporated sampling weights and design variables to ensure nationally representative estimates, following NHANES analytic guidelines.^[26,30]^

We employed weighted logistic regression to evaluate the association between log_2_(LAR) and arthritis. Within the models, log_2_(LAR) was regarded as a continuous variable. To conduct this analysis, we formulated a set of 5 distinct models: the crude model, which was unadjusted. Model 1, where adjustments were made for sex, age, race, marital status, education level, and family income. Model 2, which further accounted for smoking behavior, alcohol consumption, physical activity, CKD, cardiovascular disease (CVD), hypertension, diabetes, hyperlipidemia, and body mass index (BMI). Model 3, with additional adjustments for C-reactive protein (CRP), white blood cell count (WBC), alanine aminotransferase (ALT), and aspartate aminotransferase (AST). Model 4, where vitamin C and zinc were additionally factored into the adjustments. To investigate potential nonlinear dose–response relationships between log_2_(LAR) and arthritis, we applied a restricted cubic spline model to generate smooth curves. In this model, log_2_(LAR) was treated as a continuous variable. As recommended by Harrell, 4 knots were positioned at the 5th, 35th, 65th, and 95th percentiles.^[31]^ Nonlinearity was evaluated via a likelihood ratio test. This test involved comparing a model containing only a linear term with another model that incorporated both linear and cubic spline terms.

Additionally, we performed subgroup and interaction analyses to evaluate the consistency of the association between log_2_(LAR) and arthritis across different demographic groups. These groups were stratified according to factors such as sex, race, age, smoking status, drinking status, BMI, hypertension, and hyperlipidemia. To examine the interactions within these subgroups, we utilized likelihood ratio tests. To enhance the robustness of our analytical outcomes, we conducted several sensitivity analyses. First, we applied multiple imputation methods to address all missing covariates and subsequently repeated the analysis. Second, we segregated rheumatoid arthritis and osteoarthritis patients from the overall group of arthritis patients and conducted 2 independent analyses. Third, we excluded the outliers of the exposure factor LAR, specifically those data points below the 0.2nd percentile and above the 99.8th percentile, and reanalyzed the data. These measures were implemented to ensure the reliability and stability of our research findings.

All statistical analyses were conducted utilizing R Statistical Software (Version 4.2.2, http://www.R-project.org, The R Foundation) and the Free Statistics analysis platform (Version 2.1, Beijing, China, https://www.clinicalscientists.cn/freestatistics/).

## 3. Results

### 3.1. Baseline characteristics

Following rigorous screening using established inclusion/exclusion criteria, 8616 patients were included in this study, with an overall arthritis prevalence of 23.58%. Table 1 details participants’ general characteristics stratified by log_2_(LAR) levels. Comparative analysis revealed significant differences between higher and lower log_2_(LAR) groups: notably, the higher log_2_(LAR) group was significantly older and had a higher proportion of females, non-Hispanic Whites, married/cohabiting individuals, those with higher education levels, and participants with medium family income. This group also contained more nonsmokers, current drinkers, individuals reporting sedentary physical activity, and patients with hypertension, hyperlipidemia, diabetes, and cardiovascular disease. Additionally, they exhibited higher BMI, CRP, ALT, AST levels, and zinc intake (all *P* < .05). Conversely, no statistically significant differences were observed in WBC counts or vitamin C intake between groups (all *P* > .05).

**Table 1:** Baseline characteristics of participants in the NHANES 1999 to 2010 cycles.

Characteristic	Log_2_(LAR)					
	Total (n = 8616)	Q1 (n = 2151)	Q2 (n = 2150)	Q3 (n = 2154)	Q4 (n = 2161)	*P*-value
Sex						<.001
Male	4242 (49.48%)	1198 (54.49%)	1142 (52.56%)	1040 (47.56%)	862 (40.07%)	
Female	4374 (50.52%)	953 (45.51%)	1008 (47.44%)	1114 (52.44%)	1299 (59.93%)	
Age (yr)	44.74 (0.31)	38.87 (0.39)	43.67 (0.42)	47.32 (0.50)	51.85 (0.48)	<.001
Race						<.001
Non-Hispanic White	4195 (71.09%)	1092 (73.64%)	1105 (73.30%)	1070 (71.01%)	928 (64.32%)	
Non-Hispanic Black	1690 (11.05%)	300 (6.99%)	350 (9.27%)	423 (11.56%)	617 (18.97%)	
Mexican American	1792 (8.00%)	488 (8.81%)	454 (7.86%)	446 (8.08%)	404 (6.91%)	
Others	939 (9.86%)	271 (10.56%)	241 (9.57%)	215 (9.36%)	212 (9.81%)	
Marital status						.037
Married or living with a partner	5404 (66.24%)	1361 (65.80%)	1377 (67.24%)	1412 (68.32%)	1254 (62.96%)	
Living alone	3212 (33.76%)	790 (34.20%)	773 (32.76%)	742 (31.68%)	907 (37.04%)	
Education levels (yr)						<.001
<9	1083 (5.87%)	232 (5.31%)	245 (5.25%)	293 (6.66%)	313 (6.62%)	
9–12	3436 (37.08%)	798 (32.89%)	830 (35.04%)	886 (39.30%)	922 (43.43%)	
>12	4097 (57.05%)	1121 (61.80%)	1075 (59.71%)	975 (54.04%)	926 (49.95%)	
Family income						<.001
Low (≤ 1.3)	2514 (19.26%)	590 (17.36%)	575 (17.70%)	662 (21.56%)	687 (21.41%)	
Medium (1.3–3.5)	3340 (36.76%)	806 (35.41%)	850 (36.38%)	820 (36.06%)	864 (40.14%)	
High (> 3.5)	2762 (43.99%)	755 (47.23%)	725 (45.92%)	672 (42.38%)	610 (38.45%)	
Smoking status						<.001
Never	4556 (52.63%)	1090 (51.29%)	1112 (52.72%)	1151 (52.68%)	1203 (54.45%)	
Former	2194 (24.50%)	472 (21.17%)	551 (24.41%)	556 (24.98%)	615 (29.03%)	
Current	1866 (22.87%)	589 (27.55%)	487 (22.88%)	447 (22.34%)	343 (16.53%)	
Alcohol drinking status						<.001
Never	1159 (10.67%)	225 (9.41%)	250 (8.54%)	285 (10.62%)	399 (15.52%)	
Former	1712 (15.99%)	346 (12.34%)	389 (15.22%)	461 (16.61%)	516 (21.77%)	
Current	5745 (73.34%)	1580 (78.25%)	1511 (76.24%)	1408 (72.77%)	1246 (62.71%)	
Physical activity						<.001
Sedentary	4907 (54.15%)	1141 (51.77%)	1179 (53.26%)	1210 (52.78%)	1377 (60.60%)	
Moderate	2231 (28.71%)	626 (31.43%)	564 (27.50%)	555 (29.76%)	486 (25.00%)	
Vigorous	1478 (17.14%)	384 (16.80%)	407 (19.24%)	389 (17.45%)	298 (14.39%)	
Hypertension						<.001
No	6349 (76.94%)	1766 (84.61%)	1649 (79.22%)	1543 (74.12%)	1391 (65.75%)	
Yes	2267 (23.06%)	385 (15.39%)	501 (20.78%)	611 (25.88%)	770 (34.25%)	
Hyperlipidemia						<.001
No	2581 (31.46%)	787 (38.79%)	656 (30.54%)	566 (26.25%)	572 (28.09%)	
Yes	6035 (68.54%)	1364 (61.21%)	1494 (69.46%)	1588 (73.75%)	1589 (71.91%)	
Diabetes						<.001
No	7762 (93.35%)	1973 (94.81%)	1968 (94.67%)	1952 (93.44%)	1869 (89.26%)	
Yes	854 (6.65%)	178 (5.19%)	182 (5.33%)	202 (6.56%)	292 (10.74%)	
CVD						<.001
No	7792 (92.83%)	2030 (96.08%)	1993 (94.28%)	1904 (91.07%)	1865 (88.13%)	
Yes	824 (7.17%)	121 (3.92%)	157 (5.72%)	250 (8.93%)	296 (11.87%)	
CKD						<.001
No	8458 (99.08%)	2140 (99.67%)	2126 (99.52%)	2123 (99.18%)	2069 (97.47%)	
Yes	158 (0.92%)	11 (0.33%)	24 (0.48%)	31 (0.82%)	92 (2.53%)	
BMI (kg/m^2^)	28.55 (0.11)	26.19 (0.17)	28.16 (0.16)	29.29 (0.17)	31.69 (0.22)	<.001
CRP (mg/dL)	0.41 (0.01)	0.28 (0.01)	0.36 (0.02)	0.48 (0.03)	0.61 (0.03)	<.001
WBC (10^9^/L)	6.78 (0.04)	6.66 (0.06)	6.84 (0.06)	6.82 (0.06)	6.85 (0.06)	.25
ALT (U/L)	26.25 (0.24)	22.78 (0.34)	25.48 (0.37)	26.77 (0.42)	31.86 (1.03)	<.001
AST (U/L)	25.41 (0.23)	22.12 (0.17)	23.87 (0.22)	25.57 (0.25)	32.22 (1.04)	<.001
Vitamin C intake(mg)	85.60 (1.81)	86.59 (2.82)	82.24 (2.89)	87.49 (3.26)	86.42 (2.55)	.69
Zinc intake(mg)	12.37 (0.14)	12.61 (0.20)	12.47 (0.29)	12.51 (0.23)	11.73 (0.31)	<.001
Arthritis						<.001
No	6387 (76.42%)	1781 (85.44%)	1686 (78.93%)	1547 (72.85%)	1373 (63.84%)	
Yes	2229 (23.58%)	370 (14.56%)	464 (21.07%)	607 (27.15%)	788 (36.16%)	

ALT = alanine aminotransferase, AST = aspartate aminotransferase, BMI = body mass index, CKD = chronic kidney disease, CRP = C-reactive protein, CVD = cardiovascular disease, log_2_(LAR) = log_2_-transformed lactate dehydrogenase to albumin ratio, NHANES = National Health and Nutrition Examination Survey, WBC = white blood cells.

### 3.2. Association of LAR levels with arthritis

In our multivariable weighted logistic regression analysis, log_2_(LAR) analyzed as a continuous variable showed a significant positive association with increased arthritis risk (crude model: OR: 3.94, 95% CI: 3.20–4.84, *P* < .001; Table 2). This association persisted after adjusting for potential covariates (model 4: OR: 1.52, 95% CI: 1.16–2.01, *P* = .003; Table 2). When categorizing log_2_(LAR) into quartiles, the adjusted odds ratios for arthritis in Q2, Q3, and Q4 versus Q1 were 1.14 (95% CI: 0.93–1.39, *P* = .194), 1.22 (95% CI: 0.97–1.54, *P* = .095), and 1.37 (95% CI: 1.07–1.74, *P* = .013), respectively (model 4; Table 2). These findings indicate that higher log_2_(LAR) levels are significantly associated with greater arthritis likelihood, suggesting LAR’s potential as a disease biomarker. The linear dose–response relationship between log_2_(LAR) and arthritis in model 4 was further confirmed by weighted restricted cubic spline analysis (*P* for nonlinearity = .88; Fig. S1, Supplemental Digital Content, https://links.lww.com/MD/R578).

**Table 2:** Association between LAR and arthritis in weighted multivariable regression model.

Variable	OR (95% CI)										
	n.event/N.total	Crude model	*P*-value	Model 1	*P*-value	Model 2	*P*-value	Model 3	*P*-value	Model 4	*P*-value
Log_2_(LAR)	2229/8616	3.94 (3.20–4.84)	<.001	1.81 (1.44–2.28)	<.001	1.50 (1.17–1.91)	.002	1.52 (1.15–2.00)	.004	1.52 (1.16–2.01)	.003
Q1	370/8616	1 (Ref)		1 (Ref)		1 (Ref)		1 (Ref)		1 (Ref)	
Q2	464/8616	1.57 (1.31–1.88)	<.001	1.19 (0.99–1.44)	.065	1.13 (0.93–1.38)	.203	1.14 (0.93–1.40)	.193	1.14 (0.93–1.39)	.194
Q3	607/8616	2.19 (1.80–2.65)	<.001	1.32 (1.06–1.65)	.012	1.22 (0.97–1.53)	.091	1.22 (0.96–1.54)	.101	1.22 (0.97–1.54)	.095
Q4	788/8616	3.32 (2.78–3.97)	<.001	1.62 (1.31–2.00)	<.001	1.37 (1.09–1.72)	.009	1.36 (1.07–1.74)	.013	1.37 (1.07–1.74)	.013
*P* for trend			<.001		<.001		.009		.014		.013

Crude model was not adjusted. Model 1: adjusted for sex, age, race, marital status, education levels, family income. Model 2: adjusted for model 1 + smoking status, alcohol drinking status, physical activity, CKD, CVD, hypertension, diabetes, hyperlipidemia, and BMI. Model 3: adjusted for model 2 + CRP, WBC, ALT, AST. Model 4: adjusted for model 3 + vitamin C intake, zinc intake.

Q = quartiles, Q1: −3.426 to 1.423, Q2: 1.424–1.619, Q3: 1.620–1.835, Q4: 1.835–5.195.

ALT = alanine aminotransferase, AST = aspartate aminotransferase, CI = confidence interval, CKD = chronic kidney disease, CRP = C-reactive protein, CVD = cardiovascular disease, LAR = lactate dehydrogenase to albumin ratio, log_2_(LAR) = log_2_-transformed lactate dehydrogenase to albumin ratio, n.event = number of arthritis cases, N.total = total sample size, OR = odds ratio, Ref = reference, WBC = white blood cells.

### 3.3. Subgroup analyses

**Figure 2.**
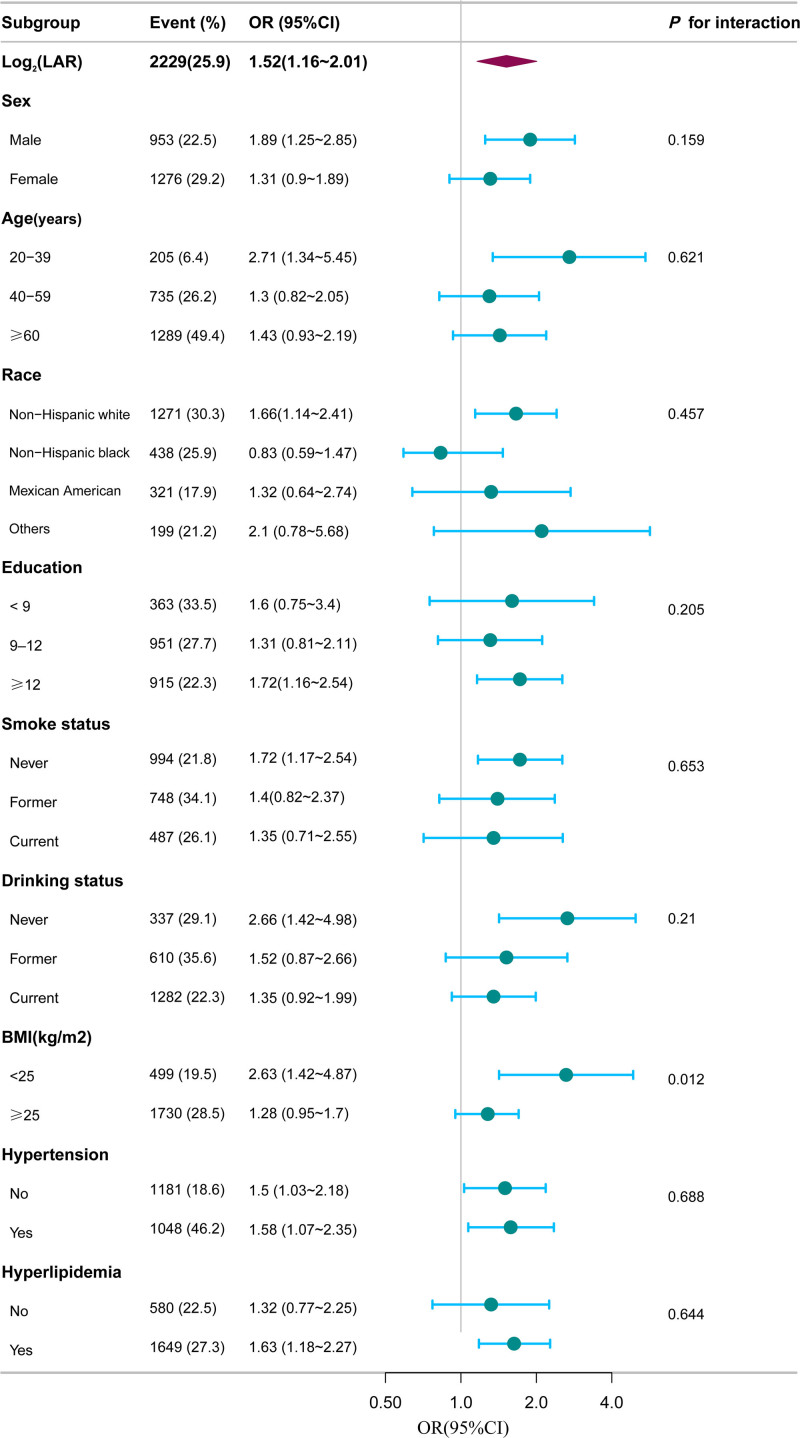
Weighted stratified and interaction analysis of the association between log_2_(LAR) and arthritis risk. Each stratification was adjusted for sex, age, race, marital status, education levels, family income, smoking status, alcohol drinking status, physical activity, CKD, CVD, hypertension, diabetes, hyperlipidemia, BMI, CRP, WBC, ALT, AST, vitamin C intake and zinc intake except for the stratification factor itself. The dots indicate odds ratios (ORs), and the horizontal lines show 95% CIs. ALT = alanine aminotransferase, AST = aspartate aminotransferase, BMI = body mass index, CI = confidence interval, CKD = chronic kidney disease, CRP = C-reactive protein, CVD = cardiovascular disease, log2(LAR) = log2-transformed lactate dehydrogenase to albumin ratio, OR = odds ratio, WBC = white blood cells.

In this study, we conducted stratified and interaction analyses to examine the consistency of the association between log_2_(LAR) and the arthritis across subgroups. No significant interactions were detected when stratifying by sex, race, age, education, smoking status, drinking status, hyperlipidemia, or hypertension (all *P*-interaction > .05). However, a significant interaction emerged in BMI stratification (*P*-interaction < .05), indicating that the association between log_2_(LAR) and arthritis varies across BMI groups, contrasting with other subgroup analyses (Fig. 2).

### 3.4. Sensitivity analysis

Sensitivity analyses results are presented in Tables S1–S4, Supplemental Digital Content, https://links.lww.com/MD/R578. First, multiple imputation for missing covariates among 10,814 participants (25.7% with arthritis, n = 2785) consistently showed higher log_2_(LAR) associated with increased arthritis risk after covariates adjustment (continuous: OR: 1.52, 95% CI: 1.16–2.01, *P* = .003; categorical Q4 vs Q1: OR: 1.37, 95% CI: 1.08–1.74, *P* = .012; Table S1, Supplemental Digital Content, https://links.lww.com/MD/R578.). Second, rheumatoid arthritis subpopulation analysis yielded similar trends (continuous: OR: 2.34, 95% CI: 1.25–4.39, *P* = .009; categorical Q4 vs Q1: OR: 1.51, 95% CI: 0.99–2.32, *P* = .057; Table S2, Supplemental Digital Content, https://links.lww.com/MD/R578). Furthermore, the analysis of the osteoarthritis subpopulation yielded the consistent trend results (Table S4, Supplemental Digital Content, https://links.lww.com/MD/R578.). Finally, after excluding extreme LAR values, model 4 confirmed the association (continuous: OR: 1.60, 95% CI: 1.21–2.13, *P* = .002; categorical Q4 vs Q1: OR: 1.36, 95% CI: 1.06–1.74, *P* = .016; Table S3, Supplemental Digital Content, https://links.lww.com/MD/R578), supporting the overall robustness of the log_2_(LAR)-arthritis relationships across all sensitivity tests.

## 4. Discussion

Our large-scale, nationally representative cross-sectional study of 8616 US adults demonstrates a significant and independent positive association between the lactate dehydrogenase to albumin ratio (LAR) and arthritis prevalence. After comprehensive adjustment for demographic, socioeconomic, lifestyle, comorbidity, inflammatory, and nutritional confounders using weighted multivariable logistic regression models, each unit increase in log_2_(LAR) was associated with a 52% higher odds of arthritis (adjusted OR: 1.52; 95% CI: 1.16–2.01; *P* = .003). Furthermore, participants in the highest quartile of log_2_(LAR) had a 37% increased odds of arthritis compared to those in the lowest quartile (adjusted OR: 1.37; 95% CI: 1.07–1.74; *P* = .013). This association remained robust across sensitivity analyses and was consistent across most subgroups, although modified by BMI. To our knowledge, this is the first study to identify LAR, a readily accessible and inexpensive biomarker integrating metabolic (lactate dehydrogenase) and inflammatory/nutritional (albumin) pathways, as an independent indicator associated with arthritis risk in the general adult population. These findings suggest LAR holds potential as a novel composite biomarker for identifying individuals at elevated risk for arthritis, particularly highlighting its relevance in metabolically unhealthy nonobese individuals, and may offer new insights into the interplay of metabolic dysregulation, inflammation, and nutritional status in arthritis pathophysiology.

The LAR has recently gained attention as a prognostic indicator for various conditions, including malignancies, liver diseases, pulmonary embolism, and infection-related complications.^[13-17]^ For instance, elevated LAR levels various poorer survival outcomes in hepatocellular carcinoma,^[32]^ non-small cell lung cancer,^[33]^ sepsis-induced acute kidney injury,^[14]^ lower respiratory infections^[17,34]^ and pulmonary embolism.^[35]^ However, its association with chronic inflammatory joint diseases remained unexplored before this study. Our findings align with emerging evidence that systemic inflammation and metabolic dysregulation contribute to arthritis progression.^[8,36]^ Specifically, lactate dehydrogenase, a key glycolytic enzyme, reflects tissue hypoxia and anaerobic metabolism. These physiological processes tend to be intensified within inflammatory microenvironments.^[9,10,37]^ On the other hand, low albumin levels may indicate poor nutrition and chronic inflammation, as albumin serves as a negative acute-phase protein with antioxidant properties.^[11,12,38]^ The interaction of these factors may worsen joint inflammation and cartilage damage, which may explain the observed association between LAR and arthritis.

The BMI-dependent effect modification observed in our study provides novel pathophysiological insights. Among non-overweight individuals, the stronger association between LAR and arthritis suggests that inflammatory and metabolic disturbances may play a more dominant role in disease pathogenesis than mechanical joint stress. Conversely, in overweight or obese individuals, the weaker link between LAR and arthritis may stem from confounding factors such as fat-related inflammation or leptin resistance, which could potentially mask LAR’s predictive value for the disease.^[39-43]^ This contrast indicates that LAR could be especially valuable as a biomarker for identifying “metabolically unhealthy non-obese” individuals at elevated arthritis risk – a population often overlooked in routine clinical assessment. To elucidate the underlying interactions, the precise biological pathways through which BMI mediates the LAR-arthritis association warrant investigation in dedicated mechanistic studies.

From a clinical perspective, LAR is a promising and accessible biomarker. Currently, arthritis management has been quite successful with various treatments. These include nonsteroidal anti-inflammatory drugs, chondroprotective agents, corticosteroids, biologics, and the new field of stem cell therapy.^[44,45]^ However, these treatments mainly focus on curing the disease or relieving symptoms after it has started or progressed. Routine measurement of LDH and albumin is cost-effective and widely available. Even in areas with limited resources, it can quickly help stratify the risk of arthritis.^[30]^ This is crucial as it allows for the early identification of high-risk individuals. With this early identification, targeted preventive measures can be taken, and public health policies can be adjusted in a timely manner before severe joint damage occurs. This approach fits well with the current precision medicine concept that emphasizes proactive identification.^[46]^ In arthritis management, monitoring LAR can supplement traditional diagnostic methods like imaging and autoantibody testing. This supplementation can help optimize treatment and inform preventive strategies. Moreover, the nutritional information from LAR values is valuable. It can guide dietary interventions to adjust albumin levels. Since albumin levels can be modified through diet, such interventions may help slow down arthritis progression or reduce the risk in vulnerable individuals.

Nevertheless, our study has several limitations. First, the arthritis diagnoses were based on self-reports from doctors, rather than standardized clinical criteria or imaging., which could lead to misclassification, especially when distinguishing between types of arthritis like osteoarthritis and rheumatoid arthritis. Second, despite employing regression models, subgroup analyses, and sensitivity analyses, we cannot rule out residual confounding from unmeasured or unidentified factors. Third, our findings are derived from a survey of American adults. Thus, their applicability to other populations is still unclear and calls for additional research. Fourth, our laboratory measurements capture only a single moment in time – assessing lactate dehydrogenase and albumin levels on a single occasion may not accurately represent ongoing, long-term exposure, potentially leading to an underestimation of the true relationships observed. Fifth, excluding certain participants reduced our sample size by 23.4%, which could introduce selection bias despite our efforts to adjust for covariates. Finally, our study’s cross-sectional nature means we cannot determine the direction of causality. It’s possible that arthritis-related inflammation could affect LAR levels, perhaps by altering liver albumin production or through the release of lactate dehydrogenase due to tissue hypoxia. Future studies should consider a longitudinal design to establish causality and explore the mechanisms linking LAR levels to arthritis more deeply.

## 5. Conclusions

In this large, nationally representative cross-sectional study of 8616 US adults, we observed a significant positive association between the lactate dehydrogenase to albumin ratio (LAR) and prevalent arthritis. After comprehensive multivariable adjustment, each unit increase in log_2_(LAR) was independently associated with 52% higher odds of arthritis (adjusted OR: 1.52; 95% CI: 1.16–2.01). Participants in the highest log_2_(LAR) quartile had 37% greater odds of arthritis compared to the lowest quartile (adjusted OR: 1.37; 95% CI: 1.07–1.74). This association was robust in sensitivity analyses and consistent across most subgroups, although modified by BMI. Our findings suggest that LAR, an inexpensive and readily available biomarker reflecting metabolic and inflammatory/nutritional status, may be a novel indicator for identifying individuals at increased arthritis risk.

## Acknowledgments

We appreciate the data collection team and NHANES staff for providing the data and reports via the NHANES website, which facilitated the creation of this article. We sincerely thank Dr Jie Liu from the Department of Vascular and Endovascular Surgery at the General Hospital of the Chinese PLA his contributions to this manuscript.

## Author contributions

**Conceptualization:** Meilin Wu, Conghai Liu.

**Data curation:** Meilin Wu.

**Formal analysis:** Meilin Wu, Meng Zheng.

**Methodology:** Meng Zheng.

**Visualization:** Meilin Wu, Zhicheng Wei.

**Writing – original draft:** Meilin Wu.

**Writing – review & editing:** Xiaodong Sun.

## Correction

This article was originally published with an incorrect reference “[18] Groenke BR, Daline IH, Nixdorf DR. SUNCT/SUNA: Case series presenting in an orofacial pain clinic. Cephalalgia. 2021;41:665–676.” The online version has now been updated with the correct reference “[18] Xie RZ, Li XS, Zhao WQ, Liang YF, Huang JF. Food insecurity and muscle health: exploring the role of protein, vitamin D, and calcium intake in low muscle mass. BMC Public Health. 2025;25:2546.”

## Supplementary Material
