# Supplementary material for: Positive association between LDH to albumin ratio and arthritis: A cross-sectional NHANES study (1999–2010)
Source: Medicine (Baltimore). 2026 Mar 27;105(13):e48096. doi: 10.1097/MD.0000000000048096 (PMC13034960; doi:10.1097/MD.0000000000048096)
Supplement: Supplementary file 1 [file medi-105-e48096-s001.docx]

**Supplementary Tables:**

**Table S1**. Associations between Log_2_(LAR) and arthritis in the multiple regression model.

| Variable | **OR (95%Cl)** | | | | | | | | | | |
| --- | --- | --- | --- | --- | --- | --- | --- | --- | --- | --- | --- |
|  | n.event/N.total | **Crude Model** | P-value | **Model 1** | P-value | **Model 2** | P-value | **Model 3** | P-value | **Model 4** | *P*-value |
| Log_2_(LAR) | 2785/10814 | 3.94(3.20-4.84) | <0.001 | 1.73(1.38-2.18) | <0.001 | 1.49(1.17-1.89) | .001 | 1.50(1.14-1.96) | .003 | 1.52(1.16-2.01) | .003 |
| Q1 | 452/2689 | 1(Ref) |  | 1(Ref) |  | 1(Ref) |  | 1(Ref) |  | 1(Ref) |  |
| Q2 | 582/2716 | 1.58(1.33-1.88) | <0.001 | 1.19(0.99-1.44) | .063 | 1.12(0.93-1.35) | .237 | 1.13(0.93-1.37) | .222 | 1.14(0.94-1.39) | .179 |
| Q3 | 760/2704 | 2.20(1.79-2.70) | <0.001 | 1.32(1.05-1.66) | .017 | 1.20(0.94-1.54) | .146 | 1.20(0.93-1.54) | .161 | 1.21(0.95-1.56) | .126 |
| Q4 | 991/2705 | 3.38(2.84-4.02) | <0.001 | 1.62(1.31-2.01) | <0.001 | 1.36(1.08-1.71) | .008 | 1.35(1.07-1.71) | .014 | 1.37(1.08-1.74) | .012 |
| *P* for Trend |  |  | <0.001 |  | <0.001 |  | .012 |  | .02 |  | .016 |

Crude Model was not adjusted. Model 1: adjusted for sex, age, race, marital status, education levelse, family income. Model 2: adjusted for Model 1 + smoking status, alcohol drinking status, physical activity, CKD, CVD, hypertension, diabetes, hyperlipidemia. BMI; Model 3: adjusted for Model 2 + CRP, WBC, ALT, AST. Model 4: adjusted for Model 3 + vitamin c intake,zinc intake.

Q = quartiles, Q1: -3.426–1.423, Q2: 1.424~1.619, Q3: 1.620~1.835, Q4: 1.835~5.195, OR = odds ratio, CI = confidence interval, Ref = reference.

**Table S2**. Association between Log_2_(LAR) and rheumatoid arthritis in weighted multivariable regression model.

| Variable | **OR (95%Cl)** | | | | | | | | | | |
| --- | --- | --- | --- | --- | --- | --- | --- | --- | --- | --- | --- |
|  | n.event/N.total | **Crude Model** | P-value | **Model 1** | P-value | **Model 2** | P-value | **Model 3** | P-value | **Model 4** | *P*-value |
| Log_2_(LAR) | 704/9261 | 4.69(3.18-6.91) | ＜0.001 | 2.45(1.55-3.87) | ＜0.001 | 2.29(1.35-3.88) | .002 | 2.37(1.27-4.39) | .007 | 2.34(1.25-4.39) | .009 |
| Q1 | 104/2270 | 1(Ref) |  | 1(Ref) |  | 1(Ref) |  | 1(Ref) |  | 1(Ref) |  |
| Q2 | 128/2358 | 1.20(0.83-1.72) | .333 | 0.91(0.62-1.34) | .645 | 0.85(0.56-1.30) | .451 | 0.85(0.56-1.31) | .462 | 0.85(0.56-1.30) | .451 |
| Q3 | 192/2313 | 2.26(1.57-3.24) | <0.001 | 1.29(0.89-1.86) | .177 | 1.16(0.77-1.73) | .473 | 1.14(0.76-1.71) | .517 | 1.11(0.74-1.66) | .607 |
| Q4 | 280/2320 | 3.96(2.89-5.43) | <0.001 | 1.87(1.26-2.77) | .002 | 1.57(1.02-2.41) | .039 | 1.52(0.99-2.33) | .056 | 1.51(0.99-2.32) | .057 |
| *P* for Trend |  |  | <0.001 |  | <0.001 |  | .013 |  | .021 |  | .023 |

Crude Model was not adjusted. Model 1: adjusted for sex, age, race, marital status, education levelse, family income. Model 2: adjusted for Model 1 + smoking status, alcohol drinking status, physical activity, CKD, CVD, hypertension, diabetes, hyperlipidemia, BMI. Model 3: adjusted for Model 2 + CRP, WBC, ALT, AST. Model 4: adjusted for Model 3 + vitamin c intake,zinc intake.

Q = quartiles, Q1: -3.426–1.427, Q2: 1.428~1.629, Q3: 1.630~1.841, Q4: 1.842~5.195, OR = odds ratio, CI = confidence interval, Ref = reference.

**Table S3**. Association between Log_2_(LAR) and arthritis in weighted multivariable regression model after outlier adjustment.

| Variable | OR (95%Cl) | | | | | | | | | | |
| --- | --- | --- | --- | --- | --- | --- | --- | --- | --- | --- | --- |
|  | n.event/N.total | **Crude Model** | P-value | **Model 1** | P-value | **Model 2** | P-value | **Model 3** | P-value | **Model 4** | *P*-value |
| Log_2_(LAR) | 2206/8530 | 4.76(3.90-5.82) | ＜0.001 | 1.90(1.49-2.43) | ＜0.001 | 1.60(1.23-2.09) | <0.001 | 1.59(1.20-2.12) | .002 | 1.60(1.21-2.13) | .002 |
| Q1 | 366/2131 | 1(Ref) |  | 1(Ref) |  | 1(Ref) |  | 1(Ref) |  | 1(Ref) |  |
| Q2 | 460/2128 | 1.58(1.31-1.91) | <0.001 | 1.19(0.98-1.45) | .084 | 1.13(0.92-1.38) | .252 | 1.13(0.92-1.39) | .244 | 1.13(0.92-1.39) | .243 |
| Q3 | 597/2132 | 2.19(1.80-2.65) | <0.001 | 1.32(1.06-1.64) | .015 | 1.21(0.96-1.52) | .102 | 1.20(0.95-1.52) | .12 | 1.21(0.96-1.52) | .113 |
| Q4 | 783/2139 | 3.38(2.83-4.04) | <0.001 | 1.62(1.32-2.01) | <0.001 | 1.37(1.08-1.73) | .01 | 1.35(1.06-1.74) | .017 | 1.36(1.06-1.74) | .016 |
| *P* for Trend |  |  | <0.001 |  | <0.001 |  | .009 |  | .017 |  | .015 |

Crude Model was not adjusted. Model 1: adjusted for sex, age, race, marital status, education levelse, family income. Model 2: adjusted for Model 1 + smoking status, alcohol drinking status, physical activity, CKD, CVD, hypertension, diabetes, hyperlipidemia, BMI. Model 3: adjusted for Model 2 + CRP, WBC, ALT, AST. Model 4: adjusted for Model 3 + vitamin c intake,zinc intake.

Q = quartiles, Q1: 0.617~1.423, Q2: 1.424~1.619, Q3: 1.620~1.834, Q4: 1.835~3.008, OR = odds ratio, CI = confidence interval, Ref = reference.

**Table S4**. Association between Log_2_(LAR) and Osteoarthritis in weighted multivariable regression model.

| Variable | **OR (95%Cl)** | | | | | | | | | | |
| --- | --- | --- | --- | --- | --- | --- | --- | --- | --- | --- | --- |
|  | n.event/N.total | **Crude Model** | P-value | **Model 1** | P-value | **Model 2** | P-value | **Model 3** | P-value | **Model 4** | *P*-value |
| Log_2_(LAR) | 824/10814 | 3.76(2.81-5.04) | ＜0.001 | 1.56(1.12-2.16) | ＜0.009 | 1.28(0.89-1.85) | .183 | 1.24(0.84-1.85) | .277 | 1.24(0.83-1.85) | .293 |
| Q1 | 116/2689 | 1(Ref) |  | 1(Ref) |  | 1(Ref) |  | 1(Ref) |  | 1(Ref) |  |
| Q2 | 163/2716 | 1.83(1.38-2.44) | <0.001 | 1.30(0.95-1.76) | .095 | 1.18(0.85-1.62) | .315 | 1.17(0.84-1.61) | .344 | 1.20(0.85-1.69) | .297 |
| Q3 | 247/2704 | 2.67(1.97-3.61) | <0.001 | 1.36(0.97-1.92) | .078 | 1.21(0.84-1.74) | .299 | 1.19(0.82-1.73) | .35 | 1.23(0.86-1.76) | .243 |
| Q4 | 298/2705 | 3.87(2.92-5.13) | <0.001 | 1.58(1.14-2.20) | .007 | 1.27(0.89-1.81) | .177 | 1.24(0.87-1.78) | .228 | 1.27(0.87-1.83) | .206 |
| *P* for Trend |  |  | <0.001 |  | .008 |  | .19 |  | .248 |  | .213 |

Crude Model was not adjusted. Model 1: adjusted for sex, age, race, marital status, education levelse, family income. Model 2: adjusted for Model 1 + smoking status, alcohol drinking status, physical activity, CKD, CVD, hypertension, diabetes, hyperlipidemia, BMI. Model 3: adjusted for Model 2 + CRP, WBC, ALT, AST. Model 4: adjusted for Model 3 + vitamin c intake,zinc intake.

Q = quartiles, Q1: -3.426–1.427, Q2: 1.428~1.629, Q3: 1.630~1.841, Q4: 1.842~5.195, OR = odds ratio, CI = confidence interval, Ref = reference.


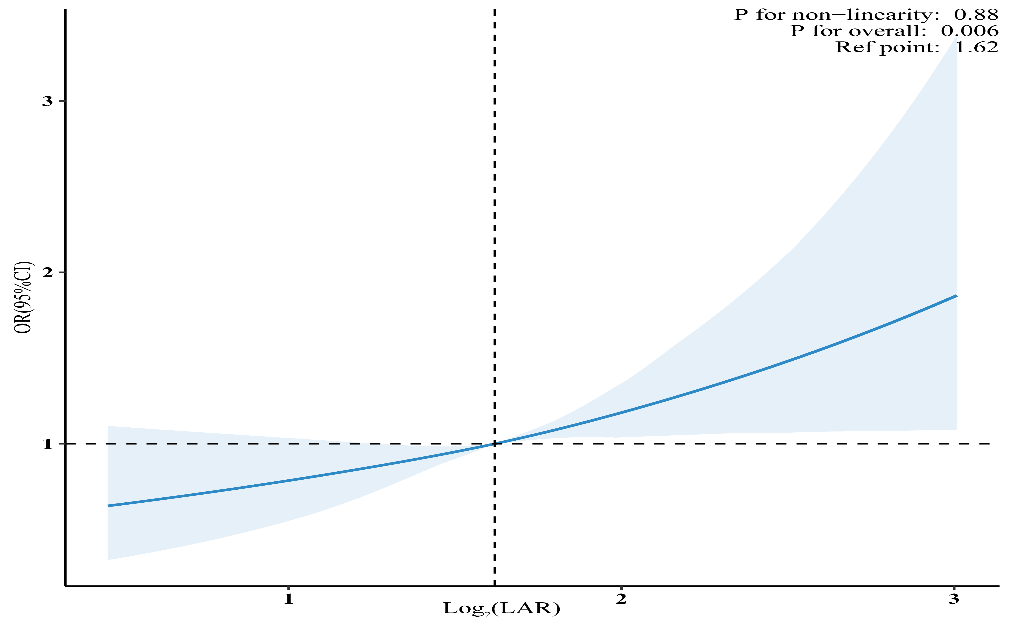
:

**Figure S1**. Restricted cubic spline model of the odds ratios of Log_2_(LAR) with arthritis risk. Solid line and shaded areas represent the predicted values and the 95% confidence intervals, respectively. They are adjusted for sex, age, race, marital status, education levels, family income, smoking status, alcohol drinking status, physical activity, CKD, CVD, hypertension, diabetes, hyperlipidemia, BMI, CRP, WBC, ALT, AST,vitamin c intake,zinc intake.
